# Supplementary material for: Overexpression of a Fragaria vesca MYB Transcription Factor Gene (FvMYB82) Increases Salt and Cold Tolerance in Arabidopsis thaliana
Source: Int J Mol Sci. 2022 Sep 11;23(18):10538. doi: 10.3390/ijms231810538 (PMC9503638; doi:10.3390/ijms231810538)
Supplement: Supplementary file 1 [file ijms-23-10538-s001.zip › Supplementary Table S1.pdf]

Table S1. List of primers used in this study.

Primer Name	Primer Sequence (5’→3’)	Purpose
AtSnRK2.4-F	GAGGAAATGGGGATGCAGAT	qPCR
AtSnRK2.4-R	TTCTCACTTCTCCACTTGCG	qPCR
AtSnRK2.6-F	AGTTGCGAGATTGATGAG	qPCR
AtSnRK2.6-R	GGCTAAATGGGTTGGTGT	qPCR
AtKUP6-F	GCAGAGACCAACGGAGAGGATGA	qPCR
AtKUP6-R	CCGGGACAACAAACCTCACTCTC	qPCR
AtNCED3-F	ATGGCTTCTTCACGGCACGG	qPCR
AtNCED3-R	TTCCTTTGCCCTCGGACG	qPCR
AtCBF1-F	CCGACTTGTTGGATAATATGGCTGAAG	qPCR
AtCBF1-R	TTAGTAACTCCAAAGCGACACGTCACC	qPCR
AtCBF2-F	CTACGAATCCCGGAATCAACCTGT	qPCR
AtCBF2-R	GCGTATAAATAGCCTCCACCAAG	qPCR
AtCOR15a-F	CAACAGAGGAATCACCAGCGA	qPCR
AtCOR15a-R	CTCTGCTGTCTTGTCGTGGTGT	qPCR
AtCOR78-F	CAACGAGGGGAAGATAAAAGTGT	qPCR
AtCOR78-R	AGCCAGATGATTTTGGAGCCT	qPCR
FvActin-F	GCGACAATGGAACTGGAATGG	qPCR
FvActin-R	GACAATTTCCCGTTCAGCAGTG	qPCR
AtActin-F	CCCGCTATGTATGTCGC	qPCR
AtActin-R	AAGGTCAAGACGGAGGAT	qPCR
FvMYB82-qF	AGCTGTAGGCTAAGGTGG	qPCR
FvMYB82-qR	ATTGTAGGCAGGCAGCAC	qPCR
FvMYB82-F	ATGGAAAACAAAAGAGTGAAAG	full-length cDNA of FvMYB82
FvMYB82-R	TCACTTATGAAATAGCTGGTAG	full-length cDNA of FvMYB82
HR-F	AGAACACGGGGGACGAGCTCATGGAAAACAAAAGAGTGAAAG	PCR for homologous recombination
HR-R	ACCATGGTGTCGACTCTAGATCACTTATGAAATAGCTGGTAG	PCR for homologous recombination
FvMYB82-slF	GTCGACATGGAAAACAAAAGAGTGAAAG	For subcellular localization
FvMYB82-slR	TCACTTATGAAATAGCTGGGGATCC	For subcellular localization
