# Supplementary figures and images for: Overexpression of a Fragaria vesca MYB Transcription Factor Gene (FvMYB82) Increases Salt and Cold Tolerance in Arabidopsis thaliana
Source: Int J Mol Sci. 2022 Sep 11;23(18):10538. doi: 10.3390/ijms231810538 (PMC9503638; doi:10.3390/ijms231810538)

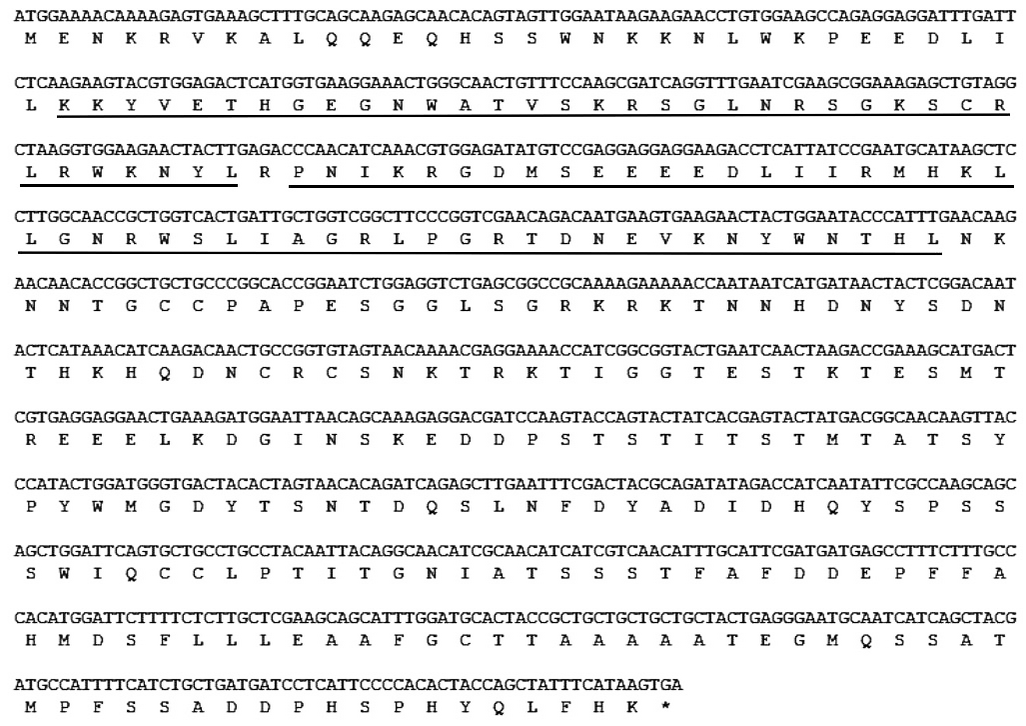

Supplement: Supplementary file 1 [file ijms-23-10538-s001.zip › Figure S1.tif]

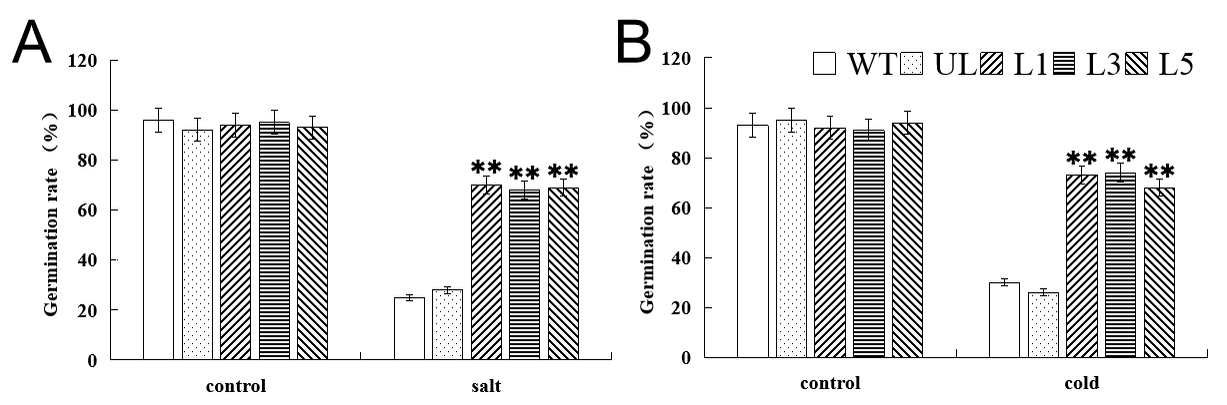

Supplement: Supplementary file 1 [file ijms-23-10538-s001.zip › Figure S2.tif]

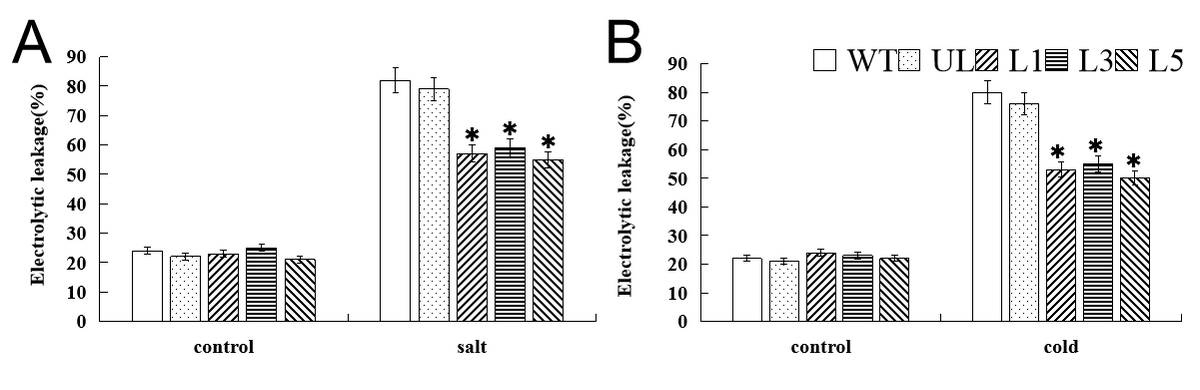

Supplement: Supplementary file 1 [file ijms-23-10538-s001.zip › Figure S3.tif]
